# Decreased Expression of Thyroglobulin and Sodium Iodide Symporter Genes in Hashimoto's Thyroiditis

**DOI:** 10.1155/2014/690704

**Published:** 2014-03-04

**Authors:** Anna Popławska-Kita, Beata Telejko, Katarzyna Siewko, Maria Kościuszko-Zdrodowska, Natalia Wawrusewicz-Kurylonek, Adam Krętowski, Justyna Hryniewicka, Janusz Dzięcioł, Witold Bauer, Robert Milewski, Małgorzata Szelachowska, Maria Górska

**Affiliations:** ^1^Department of Endocrinology, Diabetology and Internal Medicine, Medical University of Bialystok, Sklodowskiej-Curie 24A, 15-276 Bialystok, Poland; ^2^Department of Human Anatomy, Medical University of Bialystok, Mickiewicza 2A, 15-230 Białystok, Poland; ^3^Centre for Clinical Research, Medical University of Bialystok, J. Waszyngtona 15A, 15-276, Poland; ^4^Department of Statistics and Medical Informatics, Medical University of Bialystok, Szpitalna 37, 15-295 Białystok, Poland

## Abstract

*Aim*. The aim of the study was to compare the expression of sodium iodide symporter (NIS), thyroglobulin (Tg), tumor necrosis factor-**α** (TNF**α**), and interleukin-1**β** genes in patients with Hashimoto's thyroiditis (HT) and healthy individuals. *Subjects and Methods.* Thyroid cells were obtained from 39 patients with HT and 15 controls by an ultrasound guided fine needle aspiration biopsy. *Results*. The patients with HT had lower Tg and NIS mRNA (*P* = 0.002 and *P* = 0.001, resp.), as well as higher TNF**α** mRNA expression (*P* = 0.049) than the controls. In the HT group Tg mRNA expression correlated positively with NIS mRNA expression (*R* = 0.739, *P* = 0.0001) and thyroid volume (*R* = 0.465, *P* = 0.0005), as well as negatively with TNF**α** mRNA expression (*R* = −0.490, *P* = 0.001) and anti-peroxidase antibodies (TPOAb) level (*R* = −0.482, *P* = 0.0002), whereas NIS mRNA expression correlated positively with thyroid volume (*R* = 0.319, *P* = 0.02), as well as negatively with TNF**α** mRNA expression (*R* = −0.529, *P* = 0.0006) and TPOAb level (*R* = −0.422, *P* = 0.001). *Conclusions.* Our results suggest that decreased Tg and NIS expression in thyroid cells may result in reduced active iodide transport and reduced thyroid volume in patients with HT.

## 1. Introduction

Hashimoto's disease (HT) is the most common autoimmune thyroid disorder (AITD) and the most frequent cause of noniatrogenic hypothyroidism in all age groups, but its pathogenesis still remains unclear. Genetic studies have identified certain variants of human leukocyte antigen (HLA) genes, such as HLA DR3, HLA DR4, HLA DR5, HLA DQA1, DQB1, DR3/DRL, and HLA DQw7, associated with an increased risk of developing HT [1–4]. Also several other genes possibly increasing the susceptibility to AITD have been found, including the gene responsible for the expression of HLA tissue compatibility antigens located on chromosome 6, cytotoxic T-lymphocyte-associated antigen-4 (CTLA-4) located on chromosome 2, CD40 gene located on chromosome 20, the gene located on chromosome 8 encoding the expression of thyroglobulin (Tg), and the autoimmune regulator gene located on chromosome 21 [3, 4]. Furthermore, there is good evidence that certain proinflammatory cytokines, such as interleukin 1 (IL1), IL6, and tumor necrosis factor-alpha (TNF*α*), may contribute to the autoimmune disorders; however very few studies on their role in the pathogenesis of HT have been published so far [5, 6].

It is generally accepted that one of the major autoantigens involved in the development of HT is thyroglobulin [7]. Recently it was shown that Tg, stored in the follicular lumen, is a potent negative feedback regulator of physiological thyroid function that counteracts the effects of thyrotropin (TSH) [8]. In particular, Tg decreases the expression of thyroid transcription factors (TTF) which stimulate the expression of thyroid peroxidase (TPO), thyroglobulin, TSH receptor, and sodium iodide symporter (NIS) genes [9–12]. Thus, physiological concentrations of Tg significantly suppress thyroid-specific gene expression and antagonize the TSH-mediated stimulation that induces expression of thyroid-specific genes [8]. Tg coordinately regulates both basal and apical iodide transporters in thyroid follicular cells. Moreover, it was reported that Tg could induce thyroid cell growth in the absence of TSH [8, 13, 14]. In AITD thyroid cells show abnormalities in the intracellular iodine metabolism, associated with the changes in the expression of key proteins involved in the biosynthesis of TPO, Tg, NIS, and pendrin. There is good evidence that NIS synthesis in thyroid cells and its location in the cellular membrane are regulated by TSH [15, 16], but surprisingly no data on sodium iodide symporter expression in patients with HT have been published so far. Therefore, in the present study we aimed to determine the differences in the expression of thyroid-specific genes, such as NIS and Tg, as well as proinflammatory cytokines TNF*α* and IL1*β* in patients with HT and healthy individuals who had never been treated for AITD.

## 2. Subjects and Methods

### 2.1. Study Group

The group studied consisted of 53 patients with HT in clinical and hormonal euthyreosis and 28 healthy persons. The patients with HT were treated with levothyroxine for 6 months–13 years, whereas control subjects did not receive any treatment which might influence thyroid function. All individuals taking vitamins and diet supplements containing iodine were excluded. Written informed consent was obtained from all participants before enrolment, and the protocol was approved by the local ethics committee (Medical University of Bialystok).

### 2.2. Analytical Methods

Blood samples were collected from antecubital vein, between 7:30 and 8:30 a.m., after an overnight fast, in order to avoid diurnal variations. Serum TSH concentration was measured using an enzyme-linked immunoassay (DiaSource, Louvain-la-Neuve, Belgium). Anti-peroxidase antibodies (TPOAb) and TNF*α* levels were also determined by immunoassays (Euroimmun, Lubeck, Germany, and R&D Systems, Minneapolis, USA, resp.). The concentration of anti-TSH receptor antibodies was measured by a commercial radioimmunoassay (TRAK HUMAN, B-R-A-H-M-S Berlin, Germany).

### 2.3. Ultrasound Imaging and FNAB

All participants underwent thyroid ultrasonography (Aloka SSD 1100), in order to calculate thyroid volume. Then all subjects who gave the informed consent (39 patients with HT and 15 controls) underwent an ultrasound guided fine-needle aspiration biopsy (FNAB) of the thyroid. Each aspirate was smeared for conventional cytology, while the remaining part was immediately washed out of the needle with 350 mL of RLT buffer with *β*-mercaptoethanol (1 mL RLT/10 mL of *β*-mercaptoethanol) and the obtained cell material was frozen in −80°C until assayed.

### 2.4. RNA Extraction from FNAB and cDNA Synthesis

Total RNA was isolated using the RNeasy Mini Kit (Qiagen, Germany), following the manufacturer's protocol. Total RNA concentration was determined using NanoDrop ND-1000 spectrophotometer (NanoDrop 1000, THERMO Scientific, USA). cDNA synthesis was performed by High Capacity cDNA Reverse Transcription Kit (Life Technologies, USA) in the MJ Research Thermal Cycler (Model PTC-200, USA).

### 2.5. Quantitative Real-Time RT-PCR

RT-PCR was performed with TaqMan Low-Density Array chemistry (TLDA, Life Technologies, USA). The reaction mixture consisted of 5 *μ*L cDNA and 10 *μ*L of Gene Expression Master Mix (TaqMan Gene Expression Master Mix, Life Technologies, USA). Each sample was transferred to the fill port of a TaqMan Array Micro Fluidic Card and run on the 7900HT Fast Real-Time PCR System (Applied Biosystems, USA). The thermal cycling conditions included an initial activation step at 95°C for 15 min, followed by 40 cycles of denaturation, annealing, and amplification (95°C for 30 s, 55°C for 15 s, and 72°C for 30 s). The levels of Tg, NIS, TNF-*α*, and IL-1*β* transcripts were calculated after normalization of the products to the levels of glyceraldehyde-3-phosphate dehydrogenase (GAPDH) mRNA, using the 2^ΔCt^ formula, where threshold cycle (Ct) was defined as the intersection between an amplification curve and a threshold line.

### 2.6. Statistical Analysis

Statistica 10.0 for Windows (StatSoft Inc., USA) and IBM SPSS Statistics 21.0 (Predictive Solutions, USA) software were used for the statistical analysis. Before analysis data were tested for normality of distribution using Kołmogorov-Smirnov test with Lilliefors correction and Shapiro-Wilk test. Differences between the groups were compared by Mann-Whitney* U* test and relationships between variables were tested using Spearman's rank correlation test. A *P* value lower than 0.05 was considered statistically significant.

## 3. Results

The clinical and biochemical characteristics of the groups studied are shown in Table 1. As expected, the patients with HT had significantly higher concentrations of TPOAb (*P *= 0.00001) and TRAb (*P *= 0.00001), as well as higher BMI (*P *= 0.02) and smaller median thyroid volume (*P *= 0.001) than had the healthy individuals, whereas TSH values did not differ markedly between the two groups. Serum TNF*α* level was also markedly higher in the HT group in comparison with the healthy controls (*P *= 0.01). In the subjects with HT thyroid volume correlated negatively with levothyroxine dose (*R* = −0.34, *P *= 0.005). In the same group there was also a borderline correlation between thyroid volume and the treatment period (*R* = −0.24, *P *= 0.05).

As it was shown in Table 2, the patients with HT had significantly lower Tg and NIS mRNA (*P *= 0.002 and *P *= 0.001, resp.), as well as higher TNF*α* mRNA expression (*P *= 0.049) in thyroid cells than had the controls. In the HT group Tg mRNA expression correlated positively with NIS mRNA expression (*P *= 0.0001) and thyroid volume (*P *= 0.0005), as well as negatively with TNF*α* mRNA expression (*P *= 0.001) and TPOAb level (*P *= 0.0002). In the same group NIS mRNA expression correlated positively with thyroid volume (*P *= 0.02), as well as negatively with TNF*α* mRNA expression in thyroid cells (*P *= 0.0006), TNF*α* serum concentration (*P *= 0.001), and TPOAb level (*P *= 0.001). Additionally, a positive correlation between TNF*α* mRNA expression and TPOAb concentration (*P *= 0.003) was observed in the patients with HT (Table 3).

## 4. Discussion

The present study showed for the first time that NIS mRNA expression in thyroid cells was significantly lower in the patients with HT than in the healthy individuals. However no similar data concerning this thyroid-specific gene expression in patients with AITD have been published so far; our results seem to be in line with the findings of Caillou et al. [17], who demonstrated that in the subjects with lymphocytic thyroiditis follicular cells distant from lymphocytic infiltrates were negative or exhibited weak NIS immunostaining. Although in animal models of hypothyroidism an increased NIS expression was observed, possibly as a result of an excessive TSH stimulation [15, 16], its expression in the HT patients with normal TSH level seems low. Moreover, no association between NIS mRNA expression and TSH concentration was found in our study. On the other hand, negative correlations between NIS mRNA and TNF*α* mRNA expression, as well as with TNF*α* and TPOAb serum levels, might confirm the role of autoimmune and inflammatory processes in the disturbances of NIS expression in HT, even more because it was previously demonstrated that proinflammatory cytokines, such as IL1, TNF*α*, and INF*γ*, decrease NIS expression and iodine uptake in the fisher rat thyroid line-5 (FRTL-5) and in human thyrocytes [18, 19]. Furthermore, since an active iodide transport to the follicular cells is a critical step in thyroid hormone biosynthesis [20–22] and there is some evidence that NIS expression in thyroid tissue reflects the level of thyroid hormone production [23], decreased sodium iodide symporter expression seems to be an essential factor in the development of hypothyroidism in the patients with AITD.

The next finding of our study was a significant decrease in Tg mRNA expression in thyroid cells obtained from the patients with HT as compared with the healthy controls. Since recently it was shown that Tg may act as a negative feedback regulator of follicular cell function and that physiological concentrations of Tg significantly suppress thyroid-specific gene expression, antagonizing stimulatory effect of TSH, and low Tg expression might be either a compensatory mechanism or a primary defect caused by an autoimmune process. Negative associations of Tg mRNA expression with TNF-*α* mRNA expression and TPOAb level suggest the latter explanation; however the cross-sectional design of our study limits any speculations about a causal relationship between thyroid-specific gene expression and anti-thyroid antibodies. It is also worth noting that recently Suzuki et al. [8] reported that Tg could induce thyroid cell growth, even in the absence of TSH. Therefore low Tg expression in follicular cells may be responsible for smaller thyroid size in HT patients, even more because a negative correlation between Tg expression and thyroid volume was observed in the present study. We should also mention that higher TNF*α* mRNA expression and serum TNF*α* concentration in the patients with HT, as well as a positive association between TNF-*α* and TPOAb levels, might confirm a contribution of this proinflammatory cytokine to the development of AITD.

The main limitations of our study were the fact that only mRNA but not protein expression was analyzed in FNAB aspirates and that the patients with HT were treated with levothyroxine. However, since only the subjects with clinical and hormonal athyreosis were included in the study, a possible influence of an excessive TSH stimulation could be avoided.

In conclusion, our results suggest a decreased Tg and NIS expression in thyroid cells obtained from the patients with HT, which may result in a reduced active iodide transport and thyroid hormone biosynthesis, as well as reduced thyroid volume, although the question whether it is a primary defect or a compensatory mechanism needs further investigations.

## Figures and Tables

**Table 1:** Clinical and biochemical characteristics of the groups studied.

	Control group *n* = 28	Hashimoto's thyroiditis *n* = 53	*P* value
Age (years)	38.5 (25.5–54.0)	50.0 (33.5–56.5)	ns
BMI (kg/m^2^)	22.1 (20.7–27.1)	27.2 (23.9–32.1)	0.02
Thyroid volume (mL)	18.5 (12.5–19.5)	12.2 (7.9–15.3)	0.001
TSH (*μ*U/mL)	1.13 (0.92–1.37)	1.48 (1.0–1.86)	ns
TPOAb (U/mL)	6.14 (4.91–10.17)	169.7 (27.23–413.27)	0.00001
TRAb (U/L)	0.30 (0.30–0.53)	1.16 (0.82–1.35)	0.00001
TNF-*α* (pg/mL)	5.27 (4.11–6.44)	6.14 (5.27–7.62)	0.01
Levothyroxine dose (µg/day)	—	50.0 (0–88.0)	
Treatment period (years)	—	4 (2–8)	

Data are shown as medians (interquartile range); differences between groups were tested by Mann-Whitney *U* test.

**Table 2:** The expression of the genes studied in patients with Hashimoto's thyroiditis and healthy controls.

Gene	Control group *n* = 15	Hashimoto's thyroiditis *n* = 39	*P* value
NIS (AU)	0.22 (0.04–0.75)	0.12 (0.003–0.60)	0.001
Thyroglobulin (AU)	56.60 (21.34–87.96)	25.60 (1.38–20.16)	0.002
TNF-*α* (AU)	0.015 (0.002–0.03)	0.029 (0.016–0.03)	0.049
IL-1*β* (AU)	0.002 (0.0006–0.009)	0.015 (0.002–0.018)	0.26

Data are shown as medians (interquartile range); differences between groups were tested by Mann-Whitney *U* test; AU: arbitrary units.

**Table 3:** Spearman's correlation coefficients of gene expression and other variables studied in patients with Hashimoto's thyroiditis.

Variable	*R* Spearman	*P* value
Tg mRNA and thyroid volume	0.465	0.0005
Tg mRNA and NIS mRNA	0.739	0.0001
Tg mRNA and TNF*α* mRNA	−0.490	0.001
Tg mRNA and TPOAb level	−0.482	0.0002
NIS mRNA and thyroid volume	0.319	0.02
NIS mRNA and TNF*α* mRNA	−0.529	0.0006
NIS mRNA and TNF*α* level	−0.320	0.001
NIS mRNA and TPOAb level	−0.422	0.001
TNF*α* mRNA and TPOAb level	0.389	0.003
